# Effects of bias current and control of multistability in 3D hopfield neural network

**DOI:** 10.1016/j.heliyon.2023.e13034

**Published:** 2023-01-20

**Authors:** Bertrand Frederick Boui A Boya, Balamurali Ramakrishnan, Joseph Yves Effa, Jacques Kengne, Karthikeyan Rajagopal

**Affiliations:** a Unité de Recherche d’Automatique et d’Informatique Appliquée (UR-AIA), IUT-FV Bandjoun, University of Dschang, P.O. Box 134, Bandjoun, Cameroon; b Unité de Recherche de Matière Condensée, d’Electronique et de Traitement Du Signal (UR-MACETS), Department of Physics, University of Dschang, PO Box 67, Dschang, Cameroon; c Laboratory of Energy-Electric and Electronic Systems, Department of Physics, Faculty of Science, University of Yaoundé I, P.O. Box 812, Yaoundé, Cameroon; d Department of Physics, University of Ngaoundere, P.O. Box 454, Ngaoundere, Cameroon; e Center for Nonlinear Systems, Chennai Institute of Technology, India

**Keywords:** Hopfield neural networks, Bias current, Bursting oscillation, Multistability control, Microcontroller implementation

## Abstract

This work studies the dynamics of a three dimensional Hopfield neural network focusing on the impact of bias terms. In the presence of bias terms, the models displays an odd symmetry and experiences typical behaviors including period doubling, spontaneous symmetry breaking, merging crisis, bursting oscillation, coexisting attractors and coexisting period-doubling reversals as well. Multistability control is investigated by employing the linear augmentation feedback strategy. We numerically prove that the multistable neural system can be adjusted to experience only a single attractor behavior when the coupling coefficient is gradually monitored. Experimental results from a microcontroller based realization of the underlined neural system are consistent with the theoretical analysis.

## 1 Introduction

The human brain represents a complex system composed of a multitude of neurons whose study is important to understand different mechanisms of biological function in neural systems [1]. More physiological experiments show that electrical activities in biological neurons and nervous systems are closely associated with the unique abilities of the brain, including memory, thinking, and learning [2,3]. To emulate biological neural dynamics, different mathematical models of neurons have been proposed, including the Hodgkin-Huxley neural model [4], Chay neuronal model [5], Hindmarsh-Rose neuronal model [6], Morris-Lecar neural model [7], FitzHugh-Nagumo neural model [8], Hopfield neural model [9,10,11], just to name a few. Discrete Neural Network suffers more capacity issues as the number of neurons that stores pattern increases [12]. Discrete Hopfield Neural Network where the cost function of each variable was derived by minimizing the inconsistency of the logic [13,14]. Different compositions of major 2 satisfiability are implemented in discrete Hopfield neural network by adopting an exhaustive search as a training algorithm [15]. The communication between neurons can be mediated by electrical synapses across gap junctions. Despite the satisfactory results reported in these connections, many contributions have highlights the evidence for non-synaptic diffusion neurotransmission commonly referred as volume transmission in the brain [16]. In fact, the above evidence coupled with a highly plastic brain motivates research on the brain function where the non-synaptic diffusion of information transmission plays an important role [17,18]. A renewed interest in neurodynamics research based on the Hopfield neural network is the subject of several studies, in particular when neurons are exposed to external stimuli which have undergone extraordinary development. In recent years, many research works have been carried out on the study of Hopfield neural network (HNN). The spiral chaotic patterns with different dynamic amplitudes, are presented in the model [19]. Ref. [20] demonstrates that memristor synapse-based neural network with the simplest cyclic connection can exhibit chaos, coexisting attractors under different initial conditions. Chen et *al.,* explore a bidirectional coupling on two HNN neurons with an ideal flux-controlled memristor synapse. Different stability regions distributed in the parameter plane, multistability of asymmetric multi-stable patterns of the spiral chaotic patterns with different dynamic amplitudes, are demonstrated in the model [19]. Yang et *al,.* In 2021 presents a Hopfield neural network where the authors performs an analytical study and reveal interesting features such as existence of bistability, and antimonotonicity. Complex dynamics of the global coexisting multiple firing activities are explored in a bi-neurons HNN with different gradients [21]. Ref. [22] proposes a simple three dimensional Hopfield neural network model where authors performs an analytical study and report multistability of six different attractors. However, no discussion was made concerning the presence of the multistability of eight mixed attractors as well as the presence of antimonotonicity, bursting oscillations and the effect of external stimuli. Bias currents are relevant to real electrophysiological environments and can cause the complicated dynamical behaviors in neurons. Complex electrical activity of brain dynamic is usually obtained in the structure of neurons is present with external stimulus. Motivated by these interesting contributions we propose to study the Hopfield neural model with three neurons previously introduced in literature [22] with particular emphasis on the impact an external stimuli (bias current). When we consider the external stimuli, the model develop more complex dynamics such as asymmetric antimonotonicity, and asymmetric multistability. The coexistence of symmetric and asymmetric bursting oscillations in the absence and presence of bias current.

The rest of this work is organized as follows, the following section presents a theoretical study where we analyses the stability of equilibrium points. The numerical study will be discussed in Section III to show the details generated by the dynamics of this model. In Section IV, microcontroller-based implementation are performed to support our investigations. Section V concludes this paper.

### 1.1 Theoretical study

We consider the Hopfield neural networks model made of three neurons proposed in literature [22]. When the bias current is connected to the first neuron, we prove that we can control the symmetry of this model can be controlled for $I_{i}=0$, this model is odd his symmetric, the case $I_{i}\neq 0$ relates to an asymmetric system. This model is shown in equation (1),

(1) $$C_{i}\frac{dx_{i}}{dt}=-\frac{x_{i}}{R_{i}}+\sum_{j=1}^{3}w_{ij}f\left(\beta_{j}x_{j}\right)+I_{i}$$

and its simplified form in equation (2).

(2) $$\left\{ \begin{array}{l} {\dot{x}}_{1}=-x_{1}+2\;\tanh\left(\beta_{1}\;x_{1}\right)-1.2\;\tanh\left(\beta_{2}\;x_{2}\right)+0.48\;\tanh\left(\beta_{3}\;x_{3}\right)+i_{1}\\ {\dot{x}}_{2}=-x_{2}+3.6\;\tanh\left(\beta_{1}\;x_{1}\right)+1.7\;\tanh\left(\beta_{2}\;x_{2}\right)+1.076\;\tanh\left(\beta_{3}\;x_{3}\right)\\ {\dot{x}}_{3}=-x_{3}-9\;\tanh\left(\beta_{1}\;x_{1}\right) \end{array}\right.$$

Here $I_{i}={\left[i_{1},0,0\right]}^{T}$ represents the excitation current between the three neurons, $w_{ij}$ the synaptic weights between the neurons. $f\left(\beta_{j}x_{j}\right)=\tanh\left(\beta_{j}x_{j}\right)$ is the activation function of the neurons, and $\beta_{j}$ denotes the gradient of the activation function. Fixed parameters of the proposed work are describe in Table 1.

**Table 1:** Parameter of the proposed work.

Parameter	Value	Parameter	Value	Parameter	Value	Parameter	Value
$w_{11}$	2	$w_{21}$	3.6	$w_{31}$	−9	$\beta_{1}$	0.9
$w_{12}$	−1.2	$w_{22}$	1.7	$w_{32}$	0	$\beta_{3}$	1.4
$w_{13}$	0.48	$w_{23}$	1.076	$w_{33}$	0	$C_{i}=R_{i}$	1

Equilibrium state are obtained if all the derivative are equal to zero (${\dot{x}}_{1}=0$, ${\dot{x}}_{2}=0$, ${\dot{x}}_{3}=0$); hence after simple manipulation of the following equation (3),

(3) $$\left\{ \begin{array}{l} 0=-x_{1}+2\;\tanh\left(\beta_{1}\;x_{1}\right)-1.2\;\tanh\left(\beta_{2}\;x_{2}\right)+0.48\;\tanh\left(\beta_{3}\;x_{3}\right)+i_{1}\\ 0=-x_{2}+3.6\;\tanh\left(\beta_{1}\;x_{1}\right)+1.7\;\tanh\left(\beta_{2}\;x_{2}\right)+1.076\;\tanh\left(\beta_{3}\;x_{3}\right)\\ 0=-x_{3}-9\;\tanh\left(\beta_{1}\;x_{1}\right) \end{array}\right.$$

We obtain equation (4) which represents the expression of equilibrium points.

(4) $$E_{n}=\left(x_{1e}^{n},\frac{1}{\beta_{2}}arcth(\frac{10}{12}(-x_{1e}^{n}+2\;\tanh(\beta_{1}x_{1e}^{n})+0.48\;\tanh(\beta_{3}arcth(-9\;\tanh(\beta_{1}x_{1e}^{n})))+i_{1}),-9\;\tanh(\beta_{1}x_{1e}^{n})\right)$$

We determinate the numerical values of the coordinates of any points $E_{n}\left(x_{1e}^{n},x_{2e}^{n},x_{3e}^{n}\right)$ by solving graphically equation (5) under MATLAB.

(5) $$S(x_{1e})=-\frac{1}{\beta_{2}}arcth(\frac{10}{12}(-x_{1e}+2\;\tanh(\beta_{1}x_{1e})+0.48\;\tanh(\beta_{3}arcth(-9\;\tanh(\beta_{1}x_{1e})))+i_{1})+3.6\;\tanh(\beta_{1}x_{1e})+1.7\;\tanh(-\frac{1}{\beta_{2}}arcth(\frac{10}{12}(-x_{1e}+2\;\tanh(\beta_{1}x_{1e})+0.48\;\tanh(\beta_{3}arcth(-9\;\tanh(\beta_{1}x_{1e})))+i_{1}))+1.076\;\tanh(-9\;\tanh(\beta_{1}x_{1e}))$$

**Fig. 1:**
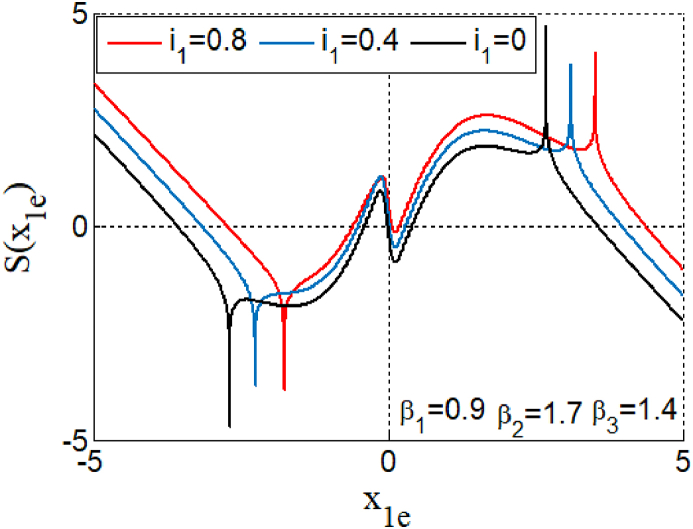
Intersection points of function curve given by Eq. (5) showing the effect of bias current on the dynamics and asymmetry equilibriums point for some value of bias current.

Considering n∈Ν the index of the equilibrium points $x_{1e}^{n}$, corresponding to the graphical intersection of the curve solutions that follows from transcendental equation (5). When we increase the bias current $i_{1}$ we see that the symmetry control parameter altered the symmetry of equilibrium points (see Fig. 1). The Jacobean matrix derived from equation (2) according to equilibrium points is given in equation (6).

(6) $$J=\left[\begin{array}{c} -2\beta_{1}\left(\tanh\;{\left(\beta_{1}x_{1}\right)}^{2}-1\right)-11.2\beta_{2}\left(\tanh\;{\left(\beta_{2}x_{2}\right)}^{2}-1\right)-0.48\beta_{3}\left(\tanh\;{\left(\beta_{3}x_{3}\right)}^{2}-1\right)\\ -3.6\beta_{1}\left(\tanh\;{\left(\beta_{1}x_{1}\right)}^{2}-1\right).7\beta_{2}\left(\tanh\;{\left(\beta_{2}x_{2}\right)}^{2}-1\right)-1-1.076\beta_{3}\left(\tanh\;{\left(\beta_{3}x_{3}\right)}^{2}-1\right)\\ 9\beta_{3}\left(\tanh\;{\left(\beta_{1}x_{1}\right)}^{2}-1\right)-1 \end{array}\right]$$

The characteristic equation associated with equation (6), is obtained from the MATLAB resolution, given in equation (7).

(7) $$\det\left(J-\lambda I_{3}\right)=\alpha_{1}\lambda^{3}+\alpha_{2}\lambda^{2}+\alpha_{3}\lambda^{1}+\alpha_{4}=0$$

By replacing the different equilibrium points in the characteristic equation, some equilibrium points are stable and the others are unstable (see Table 2) when $\beta_{1}=0.9$, $\beta_{2}=1.7$ and $\beta_{3}=1.4$ for some discrete values of bias current.

**Table 2:** Equilibrium points states, with their eigen values and stabilities for discrete value of $i_{1}$.

$i_{1}=0$	Equilibrium points $E_{0,1,2,3,4}$	Eigen values $\lambda_{1,2,3}$	Stability
	$E_{0}=\left(0,0,0\right)$	0.0182 + 0.0000i	Unstable
		−2.0541 ± 2.8082i	
	$E_{1,2}=\left(\mp 0.4023,\pm 0.0930,\pm 3.1233\right)$	−1.6199 ± 0.9119i	Stable
		−0.9959 + 0.0000i	
	$E_{3,4}=\left(\mp 3.5650,\pm 0.3941\;\pm \;0.9240i,\pm 8.9706\right)$	4.5872–0.0001i	Unstable
		−1.0066 + 0.0000i	
		−1.0000 −0.0000i	
$i_{1}=0.4$	**Equilibrium points**$E_{0,1,2,3,4}$	**Eigen values**$\lambda_{1,2,3}$	**Stability**
	$E_{0}=\left(0.0292,-0.0640,-0.2365\right)$	−0.0184 + 0.0000i	Stable
		−2.0195 ± 2.6901i	
	$E_{1}=\left(-0.5112,0.0644,3.8713\right)$	−1.6946 ± 0.8075i	Stable
		−0.9995 + 0.0000i	
	$E_{2}=\left(-3.1500,0.5464+0.9240i,8.9382\right)$	1.5633–0.0000i	Unstable
		−1.0066 + 0.0000i	
		−1.0000 + 0.0000i	
	$E_{3}=\left(0.2962,-0.1264,-2.3440\right)$	−1.5587 ± 1.0043i	Stable
		−0.9652 + 0.0000i	
	$E_{4}=\left(3.9720,-0.3144-0.9240i,-8.9859\right)$	8.2131–0.0001i	Unstable
		−1.0015 + 0.0000i	
		−1.0000 + 0.0000i	
$i_{1}=0.8$	**Equilibrium points**$E_{0,1,2,3,4}$	**Eigen values**$\lambda_{1,2,3}$	**Stability**
	$E_{0}=\left(0.0692,-0.1291,-0.5598\right)$	−1.8994 ± 2.2649i	Stable
		−0.1633 + 0.0000i	
	$E_{1}=\left(-0.4024,0.0929,3.1240\right)$	−1.6200 ± 0.9118i	Stable
		−0.9959 + 0.0000i	
	$E_{2}=\left(-2.7170,1.3248+0.9240i,8.8657\right)$	−0.8062 −0.0000i	Stable
		−1.0097 + 0.0000i	
		−1.0000 + 0.0000i	
	$E_{3}=\left(0.1914,-0.1594,-1.5352\right)$	−1.5895 ± 1.2185i	Stable
		−0.7611 + 0.0000i	
	$E_{4}=\left(4.3760,-0.2633-0.9240i,-8.9932\right)$	12.5020–0.0002i	Unstable
		−1.0007 + 0.0000i	
		−1.0000 + 0.0000i	

## 2 Numerical study

This section is devoted to the numerical analysis of the model. We present different phenomena exhibited by the model in both the symmetric and asymmetric mode of operation. Equation (2) is integrated numerically following the Runge-Kutta method adopting a constant time step Δ=0.005. The control parameter is scanned in both direction in order to uncover domains of hysteresis. Each bifurcation diagram is accompanied with related plot of Lyapunov exponent to better highlight changes in the system response as a parameter is varied under different initial condition as stacking Table 3.

1. Symmetric case ($i_{1}=0$)

**Table 3:** Methods used to obtain coexisting bifurcation diagrams of Figs. 2 and 8 and its enlargements of Figs. 2 and 8.

Figure	Control parameter range	Color	Scanning direction	Initial condition
2	$1\leq \beta_{2}\leq 2$	Green	Upward	(0, 0, 1)
	$1\leq \beta_{2}\leq 2$	Black	Upward	(0, 0, 1) fixed
	$1\leq \beta_{2}\leq 2$	Red	Upward	(0, 0, −1)
	$1\leq \beta_{2}\leq 2$	Bleu	Downward	(0, 0, −2)
3	$1\;.05\leq \beta_{2}\leq 1.24$	Green	Upward	(0, 0, 1)
	$1\;.05\leq \beta_{2}\leq 1.24$	Black	Upward	(0, 0, 1) fixed
	$1\;.05\leq \beta_{2}\leq 1.241\;.05$	Red	Upward	(0, 0, −1)
	$\leq \beta_{2}\leq 1.24$	Bleu	Downward	(0, 0, −2)
8	$1\leq \beta_{2}\leq 2$	Green	Upward	(0, 0, 1)
	$1\leq \beta_{2}\leq 2$	Black	Upward	(0, 0, 1) fixed
	$1\leq \beta_{2}\leq 2$	Red	Upward	(0, 0, −1)
	$1\leq \beta_{2}\leq 2$	Bleu	Downward	(0, 0, −2)
9	$1\;.05\leq \beta_{2}\leq 1.2$	Green	Upward	(0, 0, 1)
	$1\;.05\leq \beta_{2}\leq 1.2$	Black	Upward	(0, 0, 1) fixed
	$1\;.05\leq \beta_{2}\leq 1.2$	Red	Upward	(0, 0, −1)
	$1\;.05\leq \beta_{2}\leq 1.2$	Bleu	Downward	(0, 0, −2)

**Fig. 2:**
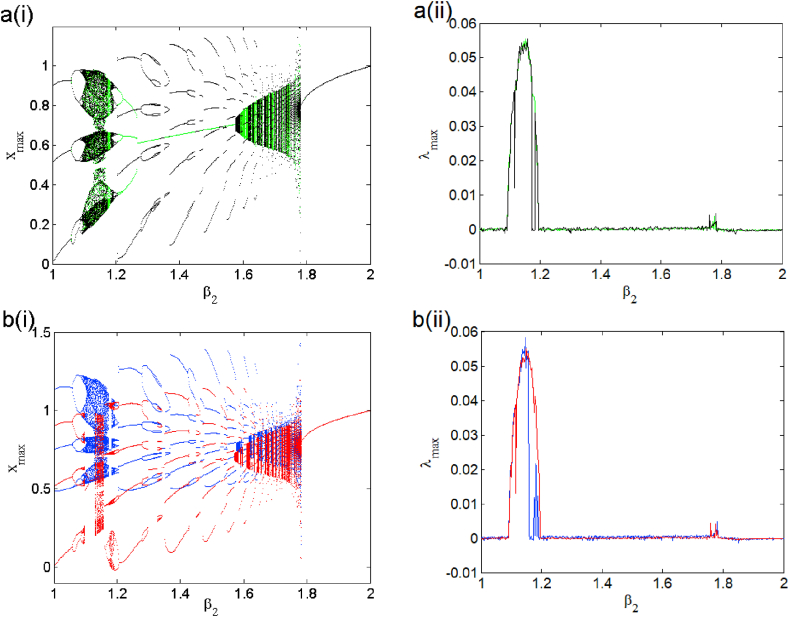
Bifurcation diagrams showing local maxima of $x_{1}$ in terms of parameter $\beta_{2}$ in a(i), b(i) and his corresponding Lyapunov spectrum.

**Fig. 3:**
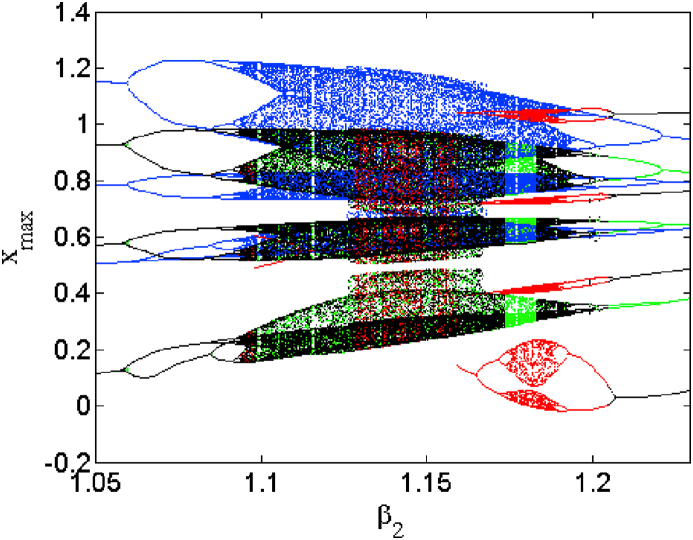
Enlargement of bifurcation diagram of Fig. 2 in the range $1.05\leq \beta_{2}\leq 1.24$.

**Fig. 4:**
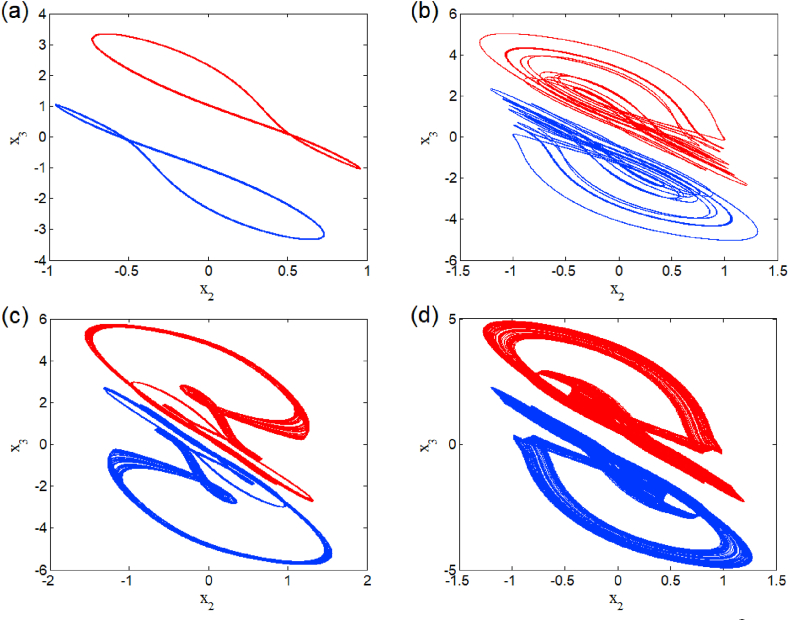
Heterogeneous multistability of eight (chaotic and periodic) attractors $\beta_{2}=1.182$ (a)–(d). The initial conditions used for these coexistence are summarized in Table 4.

**Fig. 5:**
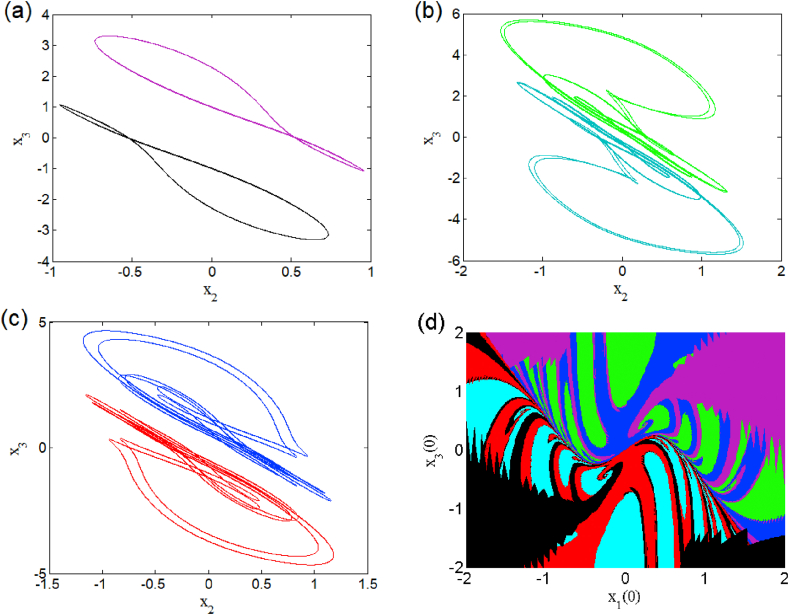
Heterogeneous multistability of six periodic attractors for $\beta_{2}=1.206$ (a)–(c). Cross section of the basins of attraction of the relevant attractors onto the plane $\left(x_{1}\left(0\right),x_{3}\left(0\right)\right)$ (d).

**Fig. 6:**
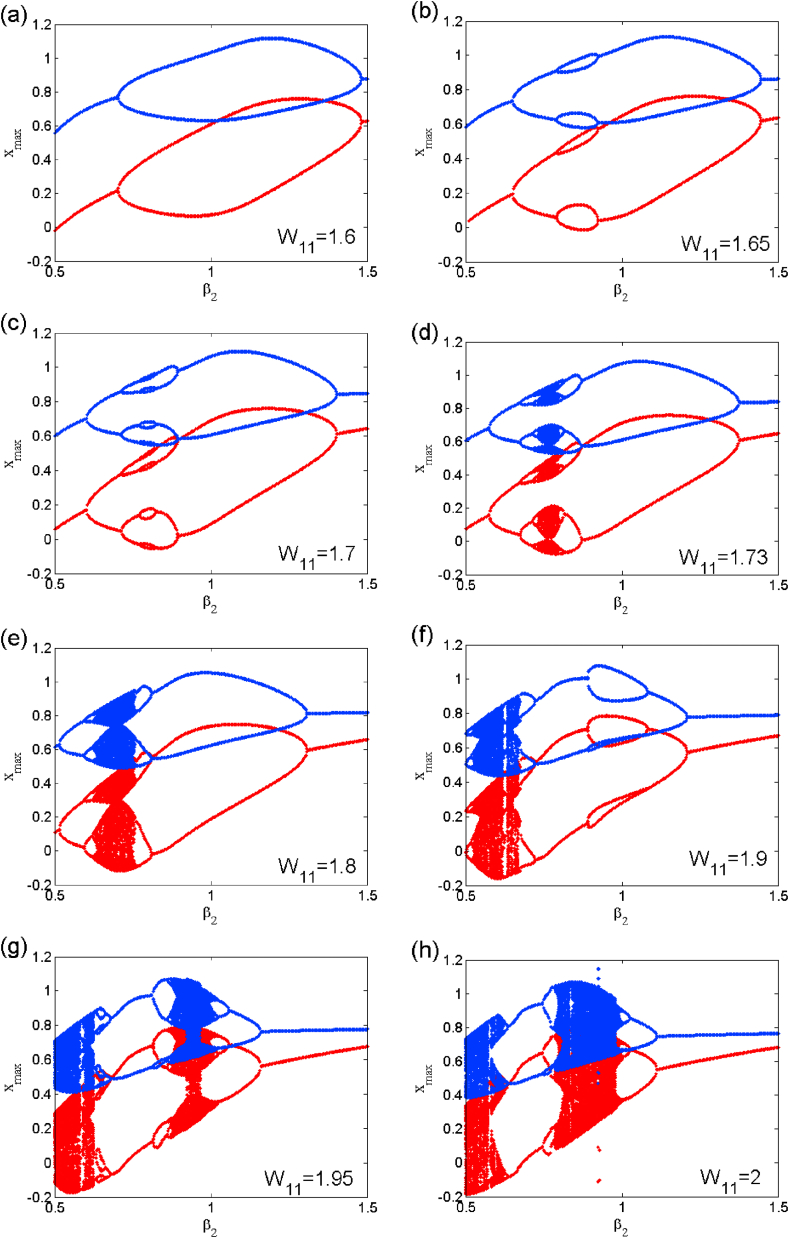
Coexisting symmetric bubbles of bifurcation obtained for several discrete values of parameter $w_{11}$ when sweeping parameter $\beta_{2}$ upward starkly from two different initial states.

**Fig. 7:**
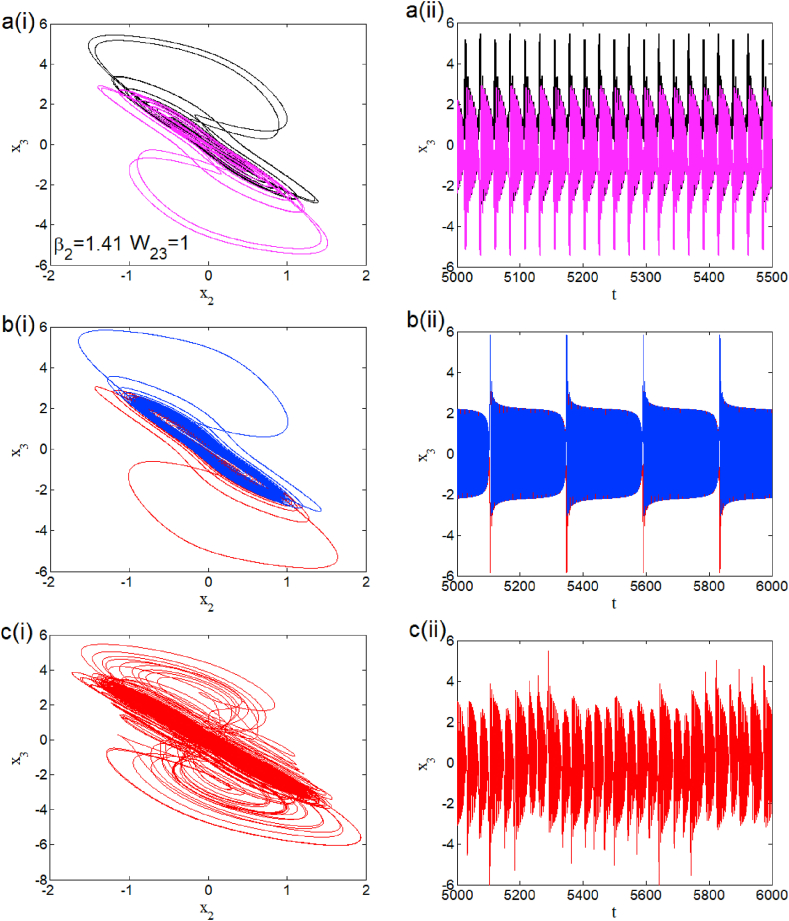
Phase portraits and time series showing the coexistence of symmetric periodic bursting for $\beta_{2}=1.41$ and $w_{23}=1$ (a), periodic bursting for a particular value of $\beta_{2}=1.78$ and $w_{11}=2$ (b) and coexistence of symmetry chaotic bursting for $\beta_{2}=1.05$ and $w_{11}=2.33$ (c).

The case $i_{1}=0$ relates to the symmetrical mode of operation. We illustrate in Fig. 2 a bifurcation diagram (i.e. Fig. 2 a(i) and b(i)) as well as their Lyapunov exponent (which verifies this bifurcation diagram) by varying parameter $\beta_{2}$ (see Fig. 2 a (ii) and b (ii))). The enlargement of these different bifurcation diagrams is presented in Fig. 3. This diagram conveys a rich and interesting dynamic such as the coexistence of multiple attractors (i.e. eight and six symmetric attractors) as shown in Fig. 4(a–d) and Fig. 5(a–c) respectively. The basin of attraction is used to present a multistable state of six different periodic limit cycles (see Fig. 5 (d) and Table 4 for more information). The route to chaos via period-bubbling is presented in Fig. 6 (a)-(h) where we clearly see the evolution of the periodic states towards the chaotic states (in red for the positive initial conditions and in blue for the negative initial conditions). For some discrete values of the parameter of gradient $\beta_{2}$ and of the synaptic weights $w_{11}$ and $w_{23}$ we notice that this system, presents periodic and chaotic bursting oscillations as displayed in phase portraits (i.e. Fig. 7 a(i), b(i) and c(i)) with their corresponding time series (see Fig. 7 a (ii), b (ii) and c (ii)).

2. Asymmetric case ($i_{1}\neq 0$**)**

**Table 4:** Coexisting multiple attractors of symmetric case for different values of $\beta_{2}$ with others parameter setting.

Value of $\beta_{2}$	Number of coexisting attractor	Initial conditions	Number of figure
1.182	8	(0, 0, ±1.32); (0, 0, ±3.46); (0, 0, ±0.21); (0, 0, ±1.09).	Fig. 4
1.183	6	(±1.44, 0, 0); (±0.5, 0, 0); (±1, 0, 0).	Not shown
1.206	6	(0, 0, ±0.21); (0, 0, ±1.05); (0, 0, ±0.5).	Fig. 5
1.636	6	(0, 0, ±0.69); (0, 0, ±0.4); (0, 0, ±1).	Not shown

**Fig. 8:**
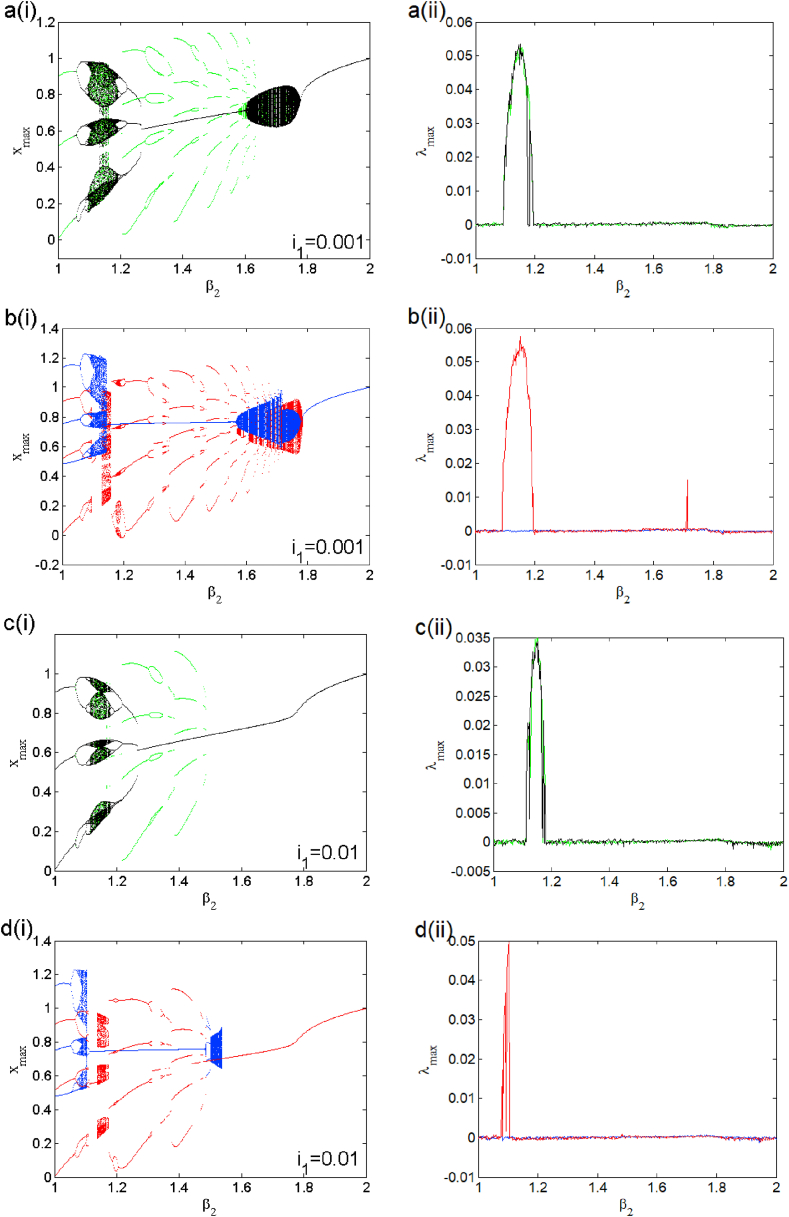
Bifurcation diagrams of shown in the left panel the local maxima of $x_{1}$ in terms of $\beta_{2}$ in a(i), b(i) for $i_{1}=0.001$ and c(i), d(i) for $i_{1}=0.01$. The corresponding Lyapunov diagrams are shown in the left pannel.

**Fig. 9:**
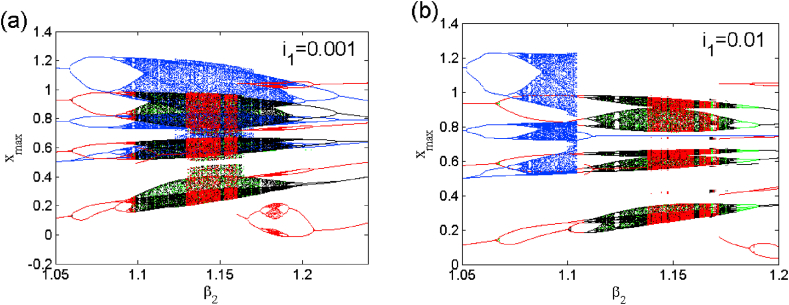
Enlargement of bifurcation diagram of Fig. 8 in the range $1.05\leq \beta_{2}\leq 1.24$

**Fig. 10:**
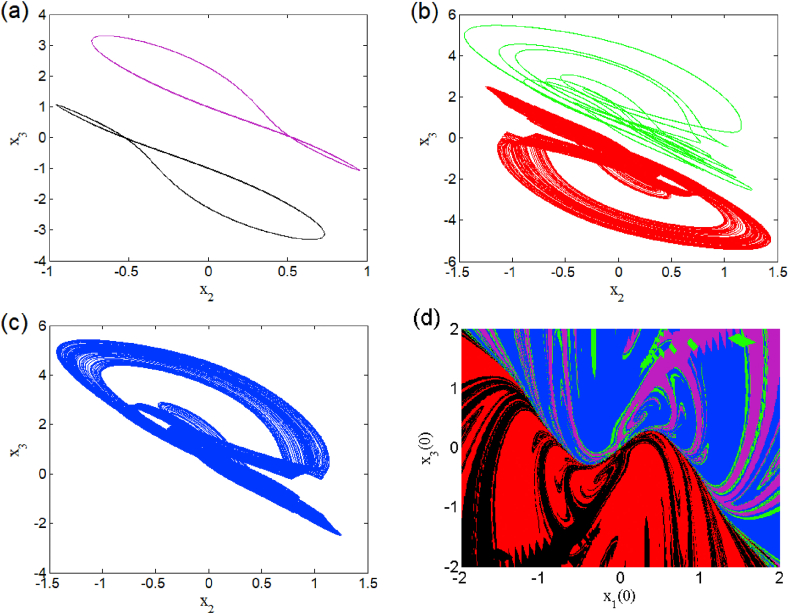
Heterogeneous multistability of five attractors for $\beta_{2}=1.114$ (a)–(c). Cross section of the basins of attraction of the relevant attractors onto the $\left(x_{1}\left(0\right),x_{3}\left(0\right)\right)$ plane provided in (d).

**Fig. 11:**
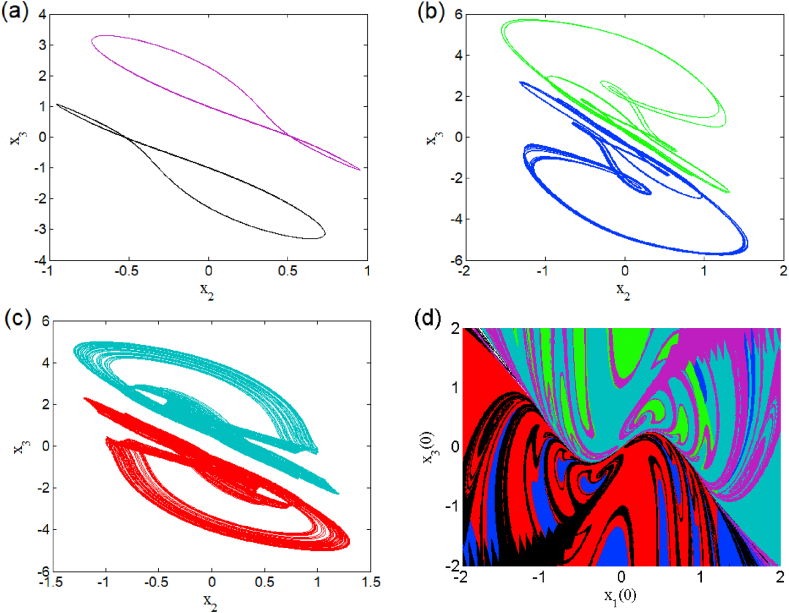
Heterogeneous multistability of six attractors for $\beta_{2}=1.173$ (a)–(c). Cross section of the basins of attraction of the relevant attractors onto the plane $\left(x_{1}\left(0\right),x_{3}\left(0\right)\right)$ (d).

**Fig. 12:**
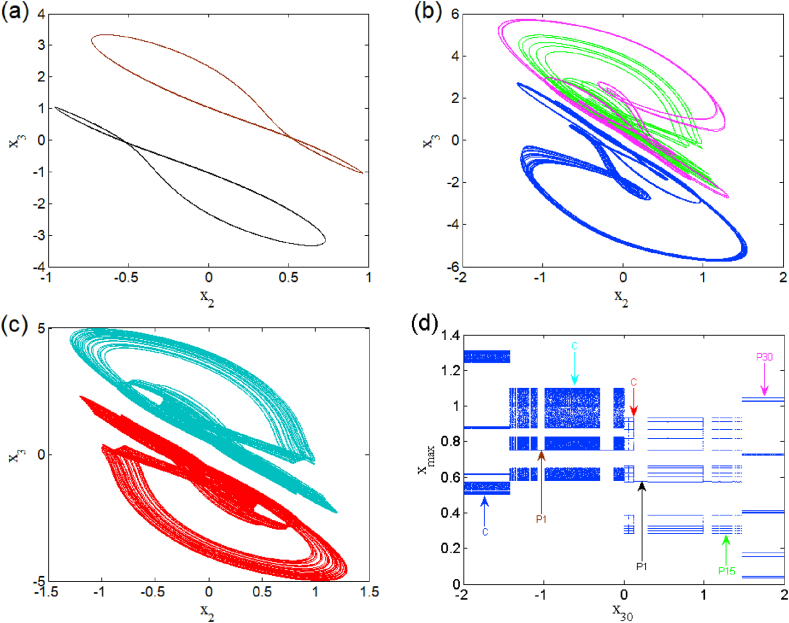
Heterogeneous multistability of seven attractors for $\beta_{2}=1.175$ (a)–(c) and 1D basins of attraction of the relevant coexisting attractors (d).

**Fig. 13:**
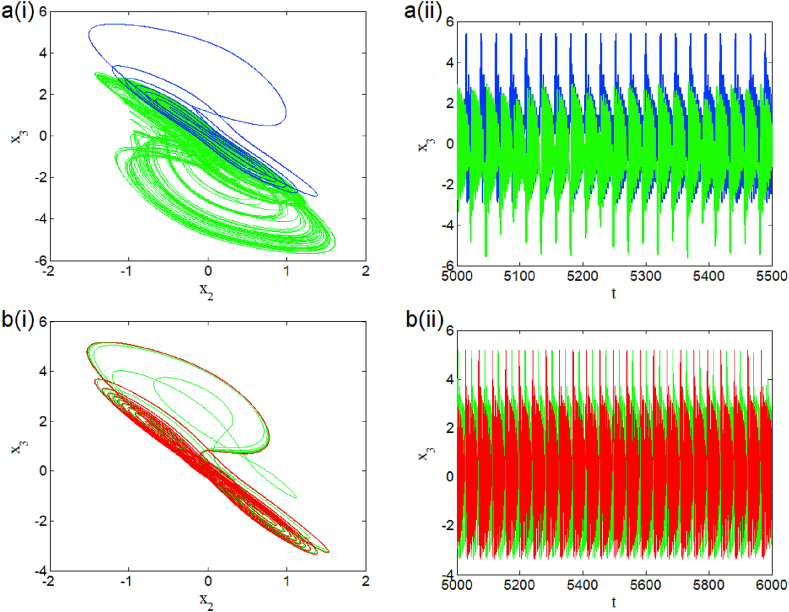
Phase portraits and time series showing coexistence of asymmetric periodic and chaotic bursting for $\beta_{2}=1.41$ and $w_{23}=1$ (a) and coexistence of asymmetric periodic bursting for $\beta_{2}=1.05$ and $w_{11}=2.33$ (b).

**Fig. 14:**
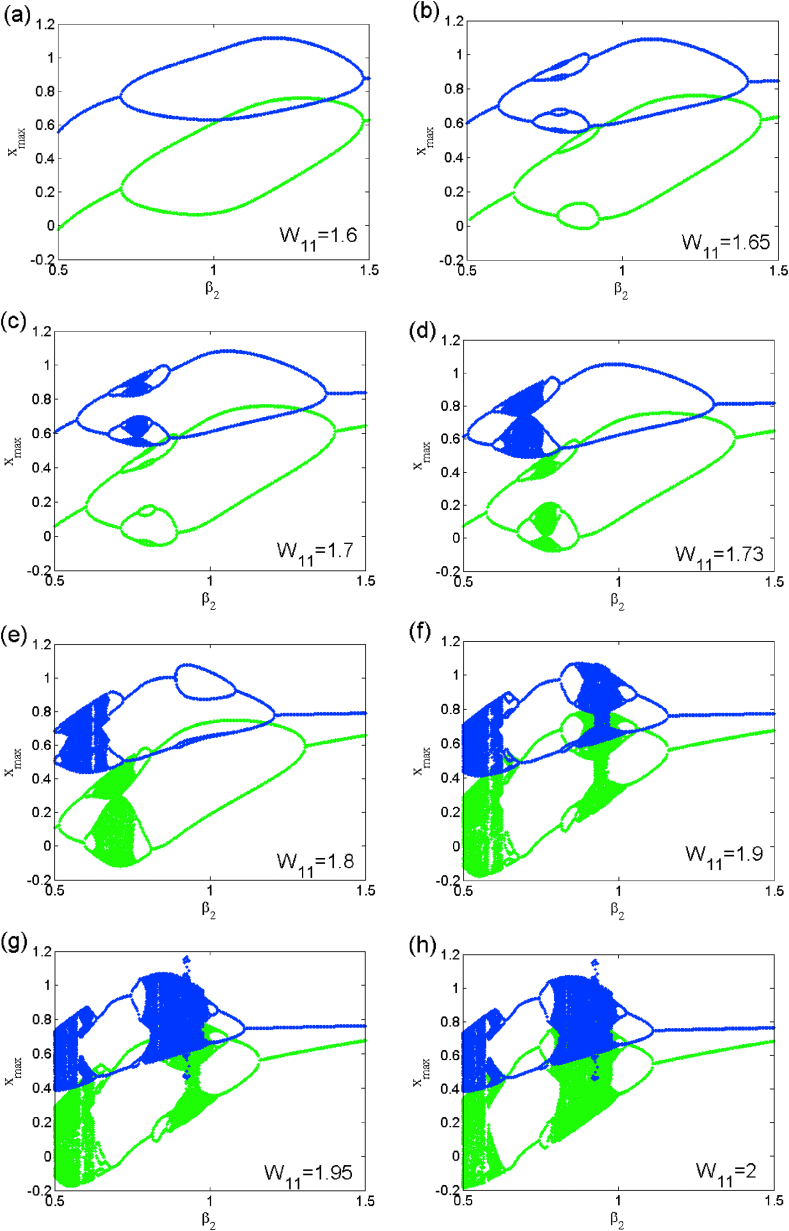
Coexisting asymmetric bubbles of bifurcation obtained for several discrete values of parameter $w_{11}$ when sweeping parameter $\beta_{2}$ upward starkly from two different initial states.

In the presence of the excitation current in ($i_{1}\neq 0$), changes the dynamic behaviors and breaks the symmetry of this system. The modified system belongs to a restricted class of dynamical whose symmetry is adjustable with a control parameter [23,24]. For two different excitation current values, we present a bifurcation diagram (see Fig. 8 a(i), b(i), c(i) and d(i)) where we highlight weak and strong symmetry breaking as well as their corresponding Lyapunov exponents (i.e. Fig. 8 a (ii), b (ii), c (ii) and d (ii)). The enlargement of these bifurcation diagrams is presented in Fig. 9 (a) and (b). We can clearly see that the symmetry breaking considerably reduces the number of coexisting attractors depending on the excitation current applied to the first neuron. This operating mode presents asymmetric oscillations such as the coexistence of multiple (e.g. five, six and seven) asymmetric attractors coexisting for $i_{1}=0.001$ (see Fig. 10, Fig. 11, Fig. 12). Basins of attraction associated to each coexisting attractors is provided onto the $\left(x_{1}\left(0\right),x_{3}\left(0\right)\right)$ plane using different colors (see Fig. 10 (d), and Fig. 11 (d)), 1D of basin of attraction is shown in Fig. 12 (d)). More information related to these coexisting attractors are given in Table 5. For $i_{1}=0.01$, we report a maximum coexistence of five different coexisting attractors. For some discrete values of the bifurcation parameter $\beta_{2}$ and synaptic weights $w_{11}$ and $w_{23}$ we notice that our model presents the coexistence of asymmetric bursting oscillations shown in phase portraits (i.e. Fig. 13 a(i) and b(i)) with their corresponding time series (see Fig. 13 a (ii) and b (ii)). The latter phenomenon makes the dynamics of this model more complex. A route to the birth of chaos via period-bubbling is displayed in Fig. 14 (a)-(h) where we clearly notice the evolution of periodic states towards chaotic states in green for positive initial conditions and in blue for negative initial conditions. The model in its asymmetric regime (perturbed) suggest that the dynamical behavior of the brain is extremely sensitive to a small external excitations.

3. Control of multistability

**Table 5:** Coexisting multiple attractors of asymmetric case for different values of $\beta_{2}$ with others parameter setting.

Value of $\beta_{2}$	Number of coexisting attractor	Initial conditions	Number of figure
1.114	5	(0, 0, ±0.42); (0, 0, ±0.18); (0, 0, 0.6).	Fig. 10
1.173	6	(0, 0, ±0.58); (0, 0, ±0.24); (0, 0, ±2).	Fig. 11
1.175	7	(0, 0, ±0.21); (0, 0, ±0.55); (0, 0, −1.5); (0, 0, 0.7); (0, 0, 2).	Fig. 12

**Fig. 15:**
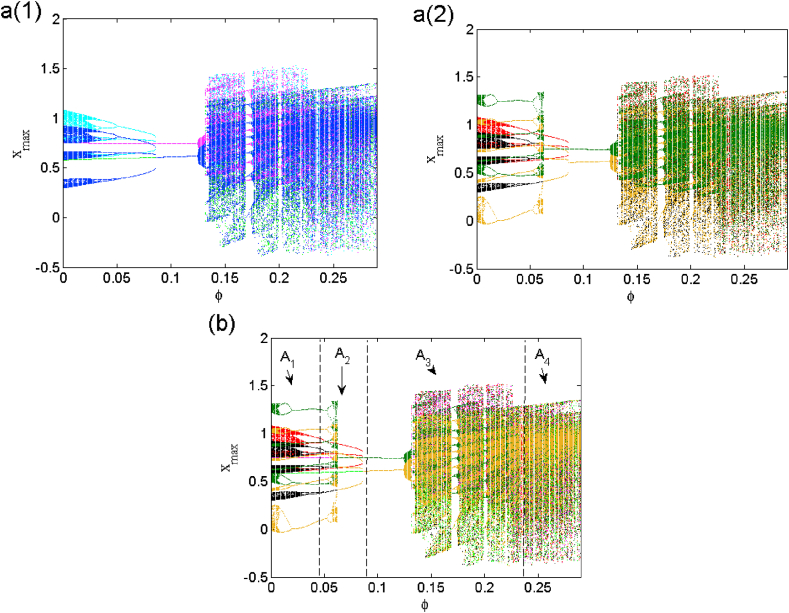
Bifurcation diagrams illustrating the control of multistability displaying the coexistence of eight solutions (see Fig. 3) to mono-stability when monitoring the coupling strength φ in 0≤φ≤0.29 area and (b) the superposition of a (1), a (2). The rest of parameters values are set as $\beta_{1}=0.9\beta_{2}=1.182$, $\beta_{3}=1.4$, ε=0.5, γ=8 and $I_{1}=0$.

Coexistence of multiple attractors offers great flexibility in the performance of dynamical system while not requiring a large change of parameter [25], and the possibility to switch between different coexisting states. However, the occurrence of different coexisting states due to small perturbations can be deplorable for the performance of dynamical model, especially by affecting its reliability and reproducibility. Moreover, this phenomenon can often lead to drawbacks in the design of a commercial device [26] with very particular characteristics. Examples of multistability showing the drawbacks of multistability in real time problems and dynamic systems are discussed in Refs. [27,28,29,30]. In this work, we perform the multistability control by linear augmentation (equation (8)) for eight symmetric attractors when $\beta_{2}=1.182$*.* This control is presented in Fig. 15 a(i)-a (ii) where we clearly see the evolution from multi-stability (area A1) to mono-stability (area A4) (see Fig. 15(b).

(8) $$\left\{ \begin{array}{l} {\dot{x}}_{1}=-x_{1}+2\;\tan\left(\beta_{1}\;x_{1}\right)-1.2\;\tan\left(\beta_{2}\;x_{2}\right)+0.48\;\tan\left(\beta_{3}\;x_{3}\right)+i_{1}\\ {\dot{x}}_{2}=-x_{2}+3.6\;\tan\left(\beta_{1}\;x_{1}\right)+1.7\;\tan\left(\beta_{2}\;x_{2}\right)+1.076\;\tan\left(\beta_{3}\;x_{3}\right)+\varphi x_{4}\\ {\dot{x}}_{3}=-x_{3}-9\;\tan\left(\beta_{1}\;x_{1}\right)\\ {\dot{x}}_{4}=-\varepsilon x_{4}-\varphi \left(x_{2}-\gamma \right) \end{array}\right.$$

## 3 Experimental investigation

**Fig. 16:**
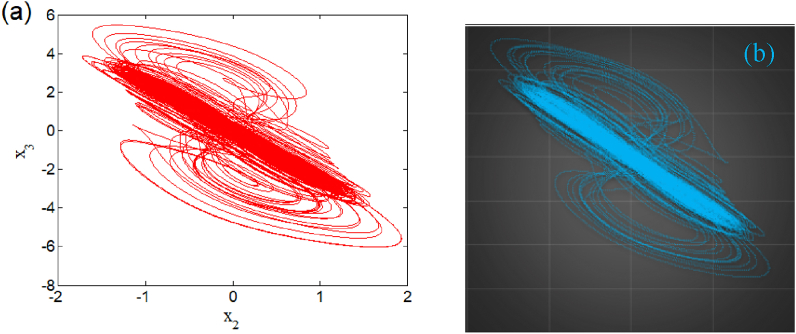
Comparaizon between numerical (a) and experimental (b) phase portraits of a chaotic bursting oscillation of the system.

**Fig. 17:**
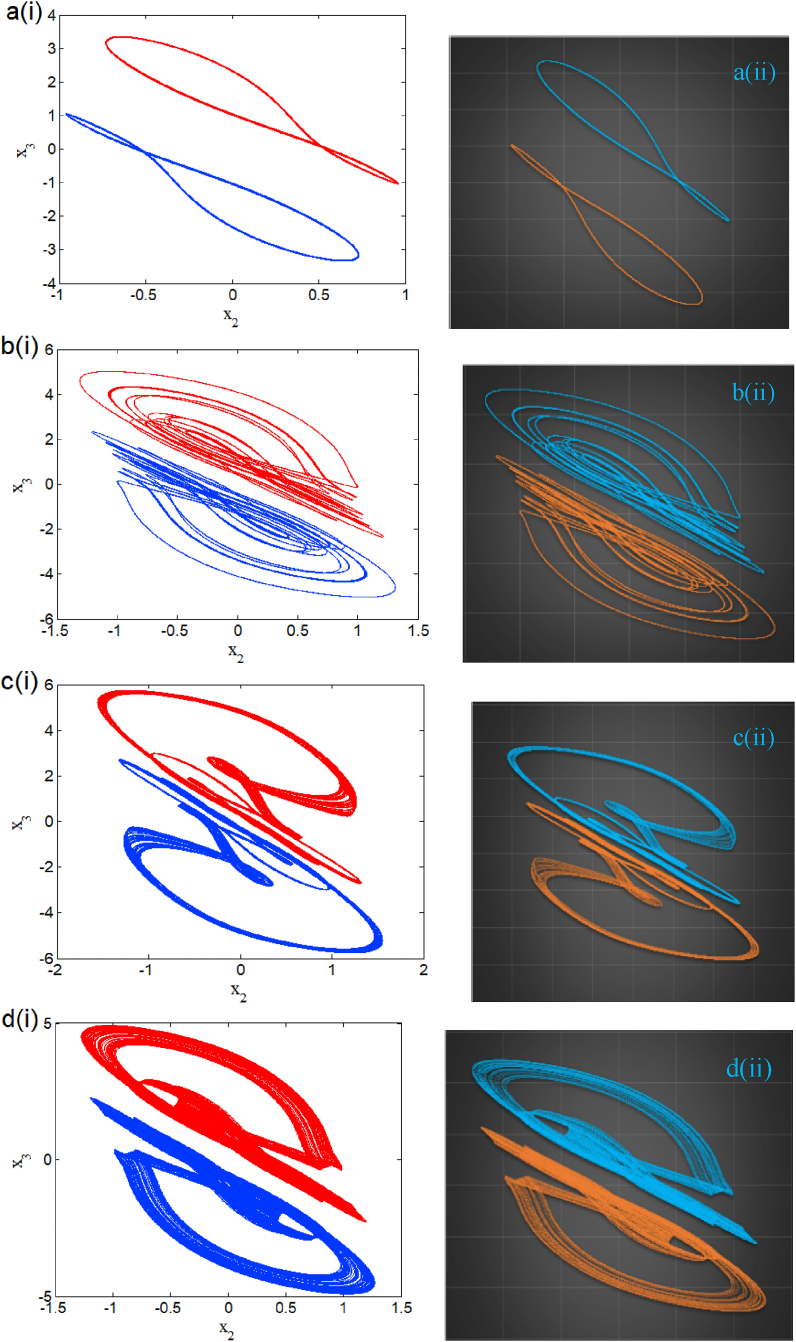
(A)–(d) Phase portraits displaying multistability of eight symmetric attractors for $\beta_{2}=1.182$ (right panel) and with it corresponding numerical plot (left panel).

The Arduino MEGA board build around the AT MEGA 2560 Microcontroller was used during the experimental phase for its speed, deep price, and accessibility. The programming language in the Arduino software IDE is C/Arduino. This language is very closed and compatible with the C programming language. Here digital components replace analog circuits as the latter is influenced by the temperature of the components, atmospheric conditions, and pressure [31,32,33,34,35], to name just a few. Based on the microcontroller implementation in described in Ref. [34], we present the phase portraits chaotic bursting phenomenon for $\beta_{2}=1.05$ and $w_{11}=2.33$ in the plane $\left(x_{2},x_{3}\right)$ with corresponding numerical result in Fig. 16(a) and the experimental device in Fig. 16(b). The coexistence of eight attractors for $\beta_{2}=1.182$ is presented microcontroller-based implementation (right panel) in Fig. 17(a)-(d) with corresponding numerical result (left panel). Based on this result, we can remark that microcontroller-based implementation results are in perfect agreement with the theoretical study.

## 4 Conclusion

The dynamical behaviors of a class of three dimensional Hopfield neural networks has been explored focusing on the effect of bias current. In case of perfect symmetry ($i_{1}=0$), the system develops various types of coexisting attractors (e.g. six or eight coexisting chaotic and periodic solutions) and the birth of chaos via period-bubbling cascades. The coexistence of symmetric bursting oscillations, hysteresis and parallel branches are also demonstrated in this system. The presence of bias current breaks the symmetry ($i_{1}\neq 0$) of the model and generates complex phenomena such as, the multistability of five, six and seven asymmetric attractors, the coexistence of asymmetric bubbles and asymmetric bursting oscillations. The linear augmentation method is used to control the multistability of the model. An experimental study based on microcontroller yields results in agreement with the theoretical analysis. The effects of symmetry breaking in neural networks is rarely reported. Hence, the results of present investigations enrich and complement previous information related to the complex dynamics of neural networks. The analysis carried out in this work was restricted to the entire order of the model, so the extension to the fractional order and discrete order represents an interesting subject for our future work.

## Author contribution statement

Bertrand Frederick Boui A Boya: Conceived and designed the experiments; Performed the experiments; Analyzed and interpreted the data; Contributed reagents, materials, analysis tools or data; Wrote the paper.

Balamurali Ramakrishnan: Conceived and designed the experiments; Analyzed and interpreted the data.

Joseph Yves Effa: Contributed to analyze and interpret the data; Wrote the paper.

Jaques Kengne: Conceived and designed the experiments; Analyzed and interpreted the data; Wrote the paper.

Karthikeyan Rajagopal: Conceived and designed the experiments; Analyzed and interpreted the data.

## Funding statement

This work is partially funded by Center for Nonlinear Systems, Chennai Institute of Technology, India vide funding number CIT/CNS/2022/RD/006.

## Data availability statement

No data was used for the research described in the article.

## Declaration of interest's statement

The authors declare no conflict of interest.
